# Prevalence and dynamics of NAFLD-associated fibrosis in people living with HIV in Vienna from first presentation to last follow-up

**DOI:** 10.1007/s00508-022-02133-9

**Published:** 2022-12-28

**Authors:** Caroline Schwarz, David Chromy, David Bauer, Nikki Duong, Victor Ulrich Schmidbauer, Michael Schwarz, Mattias Mandorfer, Armin Rieger, Michael Trauner, Michael Gschwantler, Thomas Reiberger

**Affiliations:** 1 Division of Gastroenterology & Hepatology, Department of Medicine III, Medical University of Vienna, Vienna General Hospital, Währinger Gürtel 18–20, Red Tower, Gastro-Office 7i, 1090 Vienna, Austria; 2 Vienna HIV & Liver Study Group, Medical University of Vienna, Vienna, Austria; 3 Department of Internal Medicine IV, Klinik Ottakring, Vienna, Austria; 4 Department of Dermatology, Medical University of Vienna, Vienna, Austria; 5 Department of Gastroenterology and Hepatology, Virginia Commonwealth University Medical Center, Richmond, VA USA; 6 Department of Biomedical Imaging and Image-guided Therapy, Medical University of Vienna, Vienna, Austria; 7 Rare Liver Disease (RALID) Center of the ERN RARE-LIVER, Medical University of Vienna, Vienna, Austria; 8 Sigmund Freud University, Vienna, Austria

**Keywords:** Epidemiology, Metabolic dysfunction associated fatty liver disease, Human Immunodeficiency Virus, Protease Inhibitors, Non-alcoholic Fatty Liver Disease

## Abstract

**Background/aims:**

Non-alcoholic fatty liver disease (NAFLD) is frequent in people living with HIV (PLWH) and may be aggravated by metabolic comorbidities and antiretroviral therapy (ART)-associated adverse effects.

**Methods:**

We retrospectively assessed epidemiological, clinical and laboratory parameters and ART regimens at HIV diagnosis (BL) and at last follow-up (FU) in 1458 PLWH without viral hepatitis coinfection attending our HIV clinic in 2014–2016. Fibrosis was non-invasively assessed by the NAFLD fibrosis score (NFS).

**Results:**

The median age of subjects was 37.8 years, 77.4% were male and 67.2% on ART, median CD4+ count was 356.0 cells/µL. At BL, 503 (34.5%) and 20 (1.4%) PLWH had dyslipidemia and diabetes, respectively. According to the NFS 16 (1.3%) showed advanced fibrosis (NFS ≥ 0.676), among which 1 (6.3%) had diabetes, 7 (43.8%) had dyslipidemia, and 5 (31.3%) were on HIV-protease inhibitors (PI). In addition, 191(15.1%) had intermediate NFS results, while fibrosis was ruled out (NFS ≤ 1.455) in 1065 (83.7%) PLWH.

After a median follow-up of 6.3 years, 590 (42.8%) had dyslipidemia and 61 (4.4%) had diabetes. Also, 21 (1.6%) showed advanced fibrosis, of which 10 (47.6%) had diabetes, 4 (19.0%) had dyslipidemia, and 9 (42.9%) were on PI-based ART, 223 (17.4%) had intermediate NFS results, while 1039 (81.0%) showed no fibrosis.

**Conclusion:**

During FU, advanced NAFLD fibrosis occurred in 1.3–1.6% of PLWH. Dyslipidemia, diabetes, and PI-based ART were associated with advanced NAFLD fibrosis. Prospective investigations of NAFLD severity and risk factors in PLWH are warranted.

**Supplementary Information:**

The online version of this article (10.1007/s00508-022-02133-9) contains supplementary material, which is available to authorized users.

## Introduction

Non-alcoholic fatty liver disease (NAFLD) represents one of the major causes of liver-related morbidity and mortality worldwide and affects adults as well as children [1–3]. The current global prevalence of NAFLD is estimated at 25%; however, there are pronounced differences in epidemiology according to geographical regions: While people of Hispanic origin appear to have a genetic predisposition for the development of NAFLD and therefore NAFLD prevalence is highest in South America, people of African descent are less affected by NAFLD [4]. Notably, the prevalence of non-obese NAFLD among the general population also shows a great variation between 25% or lower in some Asian countries and more than 50%, for example in Austria [5]. Recent analyses showed that the incidence of primary liver cancer as well as of cirrhosis has been increasing during the last years and, while ongoing strategies to meet the World Health Organization’s goal of viral hepatitis elimination by 2030 are in place to reduce the current burden of viral hepatitis-induced liver disease, fatty liver disease is continuously gaining importance with regard to the development of progressive liver disease and its complications [1, 2, 6–8].

Aside from genetic and/or racial predisposition, a number of risk factors have been described to be associated with the development of NAFLD [4]. While many patients who develop NAFLD during their adult life are overweight or obese, childhood obesity is also a risk factor concerning the development of NAFLD later in life [4, 9]. Furthermore, advanced age, male gender or postmenopausal status in women, western diet including high fat and high fructose consumption, and smoking represent major risk factors associated with NAFLD [10]. Consequently, metabolic disorders like type 2 diabetes mellitus (DM), dyslipidemia and cardiovascular disease are frequent in patients with NAFLD [11]. Additional factors associated with the development of NAFLD include variations in the patatin-like phospholipase domain-containing protein 3 (PNPLA3) gene, a risk allele that is most frequent in Hispanics and therefore explains some of the geographical differences in NAFLD prevalence, as well as microbiota influencing metabolic status [10, 12].

Liver disease in PLWH was traditionally mainly caused by HCV coinfection. However, with decreasing HCV prevalence and the antiretroviral therapy (ART)-attributed increased life expectancy in PLWH, liver disease will still contribute to non-HIV-related morbidity and mortality [13, 14]. Similar as for the general population, NAFLD is becoming a liver disease of growing importance among people living with HIV (PLWH) as many risk factors, like high BMI, increased waist circumference, type 2 DM, arterial hypertension and hypertriglyceridemia overlap between HIV+ and HIV− subjects [13, 15, 16]. While some studies describe a lower prevalence of moderate to severe steatosis among PLWH as compared to HIV-negative individuals [17], most analyses showed an increased risk for the development of NAFLD and non-alcoholic steatohepatitis (NASH) among PLWH of up to 50% [13–16, 18, 19].

These epidemiological discrepancies may in part be attributable to the unequal availability of ART in low and middle income countries, as ongoing HIV-induced immune activation is associated with insulin resistance, consequently triggering NAFLD [20]. On the other hand, early-generation nucleoside reverse transcriptase inhibitors and protease inhibitors are associated with the development of NAFLD and NASH [20, 21]. However, the early initiation of ART and the use of modern drugs with a favorable metabolic profile are likely limiting the contribution of these unwanted effects related to HIV and ART to NAFLD in the younger PLWH population in Austria.

As AIDS-related deaths are on the decline due to modern HIV care and ART, metabolic diseases will come to the fore among PLWH [13, 20]. NAFLD represents a relevant health risk in PLWH due to its high prevalence and the aging community of PLWH [20, 22]. While pharmacologic treatment options for NAFLD remain limited among PLWH as well as the general HIV negative population, lifestyle modification represents an effective tool to prevent the progression of fibrosis that is easy to implement and should be widely recommended [14, 15, 23]. While exercise and a Mediterranean diet generally show positive effects regarding cardiovascular risk and metabolic dysfunction, a special effort should be made in counselling PLWH at high risk for NAFLD-associated liver disease progression and/or NASH development [23–25]. As data on NAFLD in PLWH are currently scarce [13, 18, 22], we aim to provide epidemiological analyses that may serve as a basis for targeted clinical interventions and future studies.

## Patients, material and methods

### Study design and population

This retrospective data analysis was performed at the Medical University of Vienna, the largest tertiary care center in Austria and home to a capacious HIV clinic. The study cohort included all adult PLWH who attended the HIV clinic (inpatient as well as outpatient care) between January 2014 and December 2016 (36 months).

### Assessed parameters

Systematic data collection was performed using patients’ electronic medical records to extract HIV-specific parameters including HIV viral load, CD4 cell count, CDC stage and ART regimen as well as the first available viral hepatitis serology and PCR (HBV, HCV) after HIV diagnosis (defined as baseline, BL). Additionally, the following laboratory parameters were recorded at BL and at last available follow-up (FU): aspartate aminotransferase (AST), alanine aminotransferase (ALT), gamma-glutamyl transferase (GGT), alkaline phosphatase (ALP), serum bilirubin, albumin, international normalized ratio (INR), platelet count, total cholesterol, high-density lipoprotein (HDL), low-density lipoprotein (LDL) and triglycerides. The NAFLD fibrosis score (NFS) was calculated based on BL and FU parameters [26]. The body mass index (BMI) was calculated based on BL and FU height and weight, and metabolic comorbidities including dyslipidemia, diabetes and cardiovascular diseases as well as comedication were recorded. Furthermore, the main routes of HIV transmission (injection drug use, IDU; men who have sex with men, MSM; other) were determined from the HIV clinic’s electronical records.

### Fibrosis assessment and case definitions

At BL and FU, the percentage of patients with available parameters for the calculation of NFS was assessed [26] after exclusion of PLWH with:

- viral hepatitis coinfection, defined as HBsAg(+) and/or HBV-DNA(+); HCV-RNA(+) and/or
- missing or incomplete viral hepatitis serology (HBV, HCV) and/or
- missing HCV-RNA PCR in cases of anti-HCV(+).

NAFLD cases were defined as follows according to NFS at BL and at FU [26]:

- absence of advanced fibrosis (defined as F3/F4 NAFLD fibrosis): NFS < −1.455
- suspected advanced fibrosis (defined as F3/F4 NAFLD fibrosis): NFS ≥ 0.676

### Statistics

Statistical analyses were performed using IBM SPSS Statistics 27 (IBM, Armonk, NY, USA). Figures were generated using Graph Pad Prism 9.3.1 (GraphPad Software, La Jolla, CA, USA) and Microsoft Excel Version 16.58 for Mac (Microsoft, Redmond, WA, USA). Descriptive statistics including the calculation of median and interquartile range (IQR) were performed. Continuous variables were reported as median (IQR). Categorical variables were reported as number of patients with/without (proportion of patients with) the characteristic of interest. Case numbers, frequencies and percentages were calculated, and prevalence and incidence were concluded.

### Ethics statement

The study was approved by the ethics committee of the Medical University of Vienna on 7 July 2017 (EC Number: 1527/2017). The study protocol conforms to the ethical guidelines of the 1975 Declaration of Helsinki (6th revision, 2008) as reflected in a priori approval by the institution’s human research committee. Due to the retrospective character of the study, the ethics committee waived the need for written informed consent.

## Results

### Patient characteristics of the study population (Table 1)

Among 1874 PLWH who presented to our HIV clinic between 1 January 2014 and 31 December 2016, 416 (22.2%) had to be excluded due to viral hepatitis coinfection (*n* = 289) or incomplete viral hepatitis assessment (*n* = 127). A total of 1458 HIV monoinfected individuals were included in this study.

**Table 1:** Baseline characteristics of the study population

Variable	Overall	Advanced fibrosis ruled out (NFS < −1.455)	Advanced fibrosis ruled in (NFS ≥ −1.455)^a^	No NFS available
Patients (*n* (%))	**1458 (100.0)**	**1065 (73.0)**	**207 (14.2)**	**186 (12.8)**
Sex (*n* (%))				
*Male*	*1129 (77.4)*	827 (77.7)	156 (75.4)	146 (78.5)
*Female*	*329 (22.6)*	238 (22.3)	51 (24.6)	40 (21.5)
Age (years) at BL (median (IQR))	**37.8 (14.7)**	36.8 (13.6)	45.9 (17.4)	37.3 (15.9)
BMI (kg/m^2^) at BL (median (IQR))	**23.3 (4.8)**	23.0 (4.4)	25.4 (5.7)	22.3 (5.0)
Metabolic comorbidities at BL (*n* (%))				
*Diabetes mellitus*	*20 (1.4)*	5 (0.5)	14 (6.8)	1 (0.5)
*Arterial hypertension*	*96 (6.6)*	54 (5.1)	33 (15.9)	9 (4.8)
*Dyslipidemia*^b^	*503 (34.5)*	361 (33.9)	71 (34.3)	71 (38.2)
*Cardiovascular disease*	*28 (1.9)*	13 (1.3)	10 (4.8)	5 (2.7)
Route of HIV transmission (*n* (%))				
*PWID*	*83 (5.7)*	61 (5.7)	14 (6.8)	8 (4.3)
*MSM*	*778 (53.4)*	590 (55.4)	82 (39.6)	106 (57.0)
*Other*	*597 (40.9)*	414 (38.9)	111 (53.6)	72 (38.7)
ART (*n* (%))				
*No ART at BL, never ART during FU*	*110 (7.5)*	55 (5.2)	10 (4.8)	45 (24.2)
*No ART at BL, but later*	*368 (25.2)*	261 (24.5)	71 (34.3)	36 (19.4)
PI	58 (15.8)	37 (14.2)	11 (15.5)	10 (27.8)
NRTI	345 (93.8)	251 (96.2)	61 (85.9)	33 (91.7)
NNRTI	105 (28.5)	69 (26.4)	24 (33.8)	12 (33.3)
INSTI	230 (62.5)	168 (64.4)	48 (67.6)	14 (38.9)
*ART at baseline*	*980 (67.2)*	749 (70.3)	126 (60.9)	105 (56.5)
PI	356 (36.3)	260 (34.7)	61 (48.4)	35 (33.3)
NRTI	948 (96.7)	729 (97.3)	117 (92.9)	102 (97.1)
NNRTI	417 (42.6)	315 (42.1)	43 (34.1)	59 (56.2)
INSTI	206 (21.0)	169 (22.6)	30 (23.8)	7 (6.7)
*Time on ART (months) (median (IQR))*^c^	*85.0 (101.0)*	79.0 (86.0)	99.0 (92.0)	134.5 (191.0)
HIV-RNA at BL				
*viral load (log*_*10*_*/ml) (median (IQR))*	*4.6 (1.7)*	4.7 (1.6)	4.7 (1.5)	4.5 (3.8)
*HIV-RNA suppression (<* *1.7 log*_*10*_*/ml) (n (%))*	*221 (15.2)*	151 (14.2)	24 (11.6)	46 (24.7)
*CD4 count at BL (cells/µL) (median (IQR))*	*356.0 (327.3)*	362.0 (329.8)	327.0 (306.0)	353.0 (371.0)
*AIDS at BL (n (%))*	*387 (26.5)*	265 (24.9)	67 (32.4)	55 (29.6)
Laboratory parameters at BL (median (IQR))				
*AST (U/l)*	*27.0 (12.0)*	26.0 (11.0)	30.0 (20.0)	26.0 (14.0)
*ALT (U/l)*	*26.0 (19.0)*	26.0 (20.0)	23.0 (19.0)	27.0 (20.0)
*GGT (U/l)*	*30.0 (35.0)*	28.0 (31.0)	36.0 (48.0)	33.5 (35.0)
*Bilirubin (mg/dl)*	*0.5 (0.3)*	0.5 (0.3)	0.5 (0.3)	0.5 (0.3)
*Platelet count (G/l)*	*214.0 (77.0)*	223.0 (73.0)	168.0 (66.0)	215.0 (94.0)
*Total cholesterol (mg/dl)*	*183.0 (61.0)*	181.5 (58.0)	176.0 (61.3)	202.0 (74.0)
*HDL (mg/dl)*	*43.0 (17.0)*	44.0 (17.0)	41.0 (17.5)	44.0 (15.0)
*LDL (mg/dl)*	*109.0 (45.0)*	109.0 (44.0)	107.0 (41.5)	120.0 (45.0)
*Triglycerides (mg/dl)*	*123.5 (106.0)*	119.0 (97.5)	137.5 (113.3)	163.0 (148.5)

*NFS* NAFLD-fibrosis score, *BL* baseline, *IQR* interquartile range, *BMI* body mass index, *HIV* human immunodeficiency virus, *PWID* people who inject drugs, *MSM* men who have sex with men, *ART* antiretroviral therapy, *FU* follow-up, *PI* protease inhibitor, *NRTI* nucleoside reverse transcriptase inhibitor, *NNRTI* non-nucleoside reverse transcriptase inhibitor, *INSTI* integrase inhibitor, *RNA* ribonucleic acid, *AIDS* acquired immunodeficiency syndrome, *AST* aspartate aminotransferase, *ALT* alanine aminotransferase, *GGT* gamma-glutamyl transferase, *HDL* high-density lipoprotein, *LDL* low-density lipoprotein

^a^ including *n* = 16 (1.2%) patients with suspected advanced fibrosis according to NFS ≥ 0.676

^b^ dyslipidemia was defined according to the European Society of Cardiology (ESC) 2016 guidelines, using an LDL cut-off of > 190 mg/dl. Patients already treated with a lipid-lowering agent at BL were classified as dyslipidemic

^c^ by the time of data cut (17 June 2017)

The study population comprised 1129 (77.4%) male and 329 (22.6%) female PLWH with a median age of 37.8 (IQR 14.7, 45.5–30.8) years at BL. In 83 (5.7%) the suspected route of HIV transmission was IDU, 778 (53.4%) were MSM and 597 (40.9%) reported other routes of transmission: 511 (35.0%) indicated heterosexual transmission, 14 (1.0%) had previously received blood transfusions, 5 (0.3%) had a history of coagulopathy, 5 (0.3%) had nosocomial HIV infections (e.g. needlestick injuries, contaminated surgical equipment, dental interventions), 1 (0.1%) was a case of vertical HIV transmission, and for 61 (4.2%) patients the route of HIV transmission remained unclear. Most patients (*n* = 1135; 77.8%) were of European descent, while 170 (11.7%) came from Africa, 54 (3.7) from the Middle East, 48 (3.3%) from Asia, and 37 (2.5%) from South America.

While at BL 221 (15.2%) showed HIV-RNA suppression, defined as an HIV-RNA viral load < 1.7log_10_ copies/ml, 980 (67.2%) were started on ART directly at BL. Among 1348 (92.5%) PLWH who received ART throughout the overall follow-up period (individual BL date until data cut-off at 17 June 2017), 414 (30.7%) were treated with PI-containing ART regimens. 1184 (81.2%) of PLWH presented at CDC stage A; however, 387 (26.5%) already showed AIDS-defining CD4+ T‑lymphocyte counts < 200 cells/µl.

### Metabolic profile of the study population at BL (Table 1)

Among 1400 (96.0%) PLWH for whom BMI was available at BL, 347 (24.8%) were overweight (defined as BMI 25.0–29.9 kg/m^2^), 78 (5.6%) and 17 (1.2%) and 4 (0.3%) presented with obesity grade I (defined as BMI 30.0–34.9 kg/m^2^) and grade II (defined as BMI 35.0–39.9 kg/m^2^) and grade III (defined as BMI ≥ 40.0 kg/m^2^), respectively [27]. A total of 872 (62.3%) individuals showed a normal weight (defined as BMI 18.5–24.9 kg/m^2^) and 82 (5.9%) were underweight (defined as BMI < 18.5 kg/m^2^). Dyslipidemia was present in 506 (34.7%) subjects at BL, 96 (6.6%) had arterial hypertension, 20 (1.4%) had DM and 158 (10.8%) showed hyperuricemia (defined as serum uric acid ≥ 6.5 mg/dl for females and ≥ 7.0 mg/dl for males) [28], 21 (1.4%) PLWH suffered from cardiovascular disease and 7 (0.5%) from cerebrovascular disease at BL.

### Prevalence of NAFLD and suspected advanced fibrosis at BL (Table 1; Fig. 1)

At BL, NFS was available for 1272/1458 (87.2%) PLWH: 1065 (83.7%) showed NFS-Scores < −1.455, hence advanced fibrosis was ruled out in these individuals. 16 (1.3%) had NFS-Scores ≥ 0.676, suggestive of advanced fibrosis. In 191 (15.1%) subjects, intermediate NFS-Scores between ≥ −1.455 and < 0.676 were calculated.

**Fig. 1:**
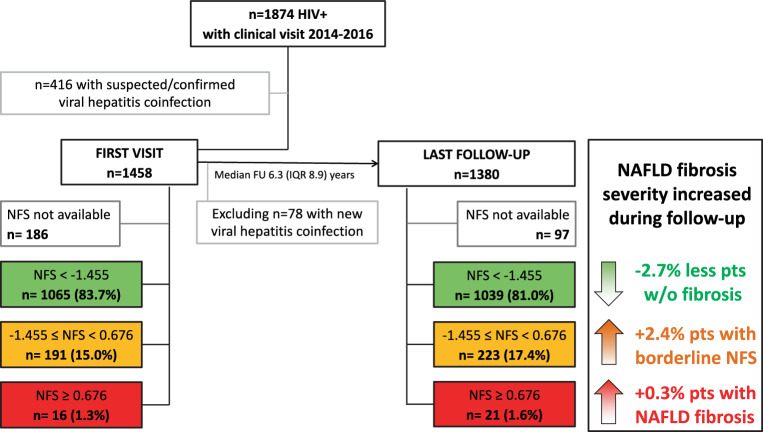
Flowchart of the study population. Assessment of NAFLD-associated advanced fibrosis according to NAFLD fibrosis score (NFS). Percentages were calculated for the subgroup of patients with available NFS. *HIV* human immunodeficiency virus, *HBV* hepatitis B virus, *HCV* hepatitis C virus, *NFS* NAFLD fibrosis score, *FU* follow-up

Among the 16 PLWH with suspected advanced fibrosis, metabolic comorbidities were prevalent in 11 (68.8%) at BL (BMI ≥ 25.0 kg/m^2^: *n* = 8 (50.0%), dyslipidemia: *n* = 7 (43.8%), DM: *n* = 1 (6.25%), hyperuricemia: *n* = 2 (12.5%), arterial hypertension: *n* = 2 (12.5%), cardiovascular disease: *n* = 1 (6.25%)). Additionally, 4 (25.0%) individuals showed serum HDL cholesterol levels below recommended cut-offs (defined as serum HDL < 50 mg/dl for females and < 40 mg/dl for males), 2 of which (12.5%) also had serum triglyceride levels > 150 mg/dl [29]. No cases of cerebrovascular disease were recorded among patients with suspected advanced fibrosis at BL. However, 5/16 (31.3%) subjects received PI-containing ART regimens.

Importantly, 6 (37.5%) of the PLWH with suspected advanced fibrosis showed normal AST, ALT, and GGT (defined as < 35 U/ml for females and < 50 U/ml for males).

### Metabolic profile of the study population at FU

During a median FU period of 6.3 (IQR 8.9) years, 78/1458 (5.3%) PLWH included in the study acquired viral hepatitis coinfection and were therefore excluded from the FU analyses.

FU BMI was available for 1303 (94.4%) of 1380 subjects included in the FU analyses: 394 (30.2%) were overweight, 129 (9.9%) showed obesity I°, 36 (2.8%) presented with obesity II°, and obesity III° was prevalent in 12 (0.9%). Of the patients 59 (4.5%) were underweight, while 673 (51.7%) showed a BMI within the normal range. Metabolic comorbidities were distributed as follows among the 1311/1380 (95.0%) PLWH for whom FU data were available: 590 (42.8%) had dyslipidemia, 351 (26.8%) suffered from arterial hypertension, 61 (4.7%) had been diagnosed with DM, 147 (11.2%) showed hyperuricemia, 65 (5.0%) had cardiovascular disease and 9 (0.7%) were affected by cerebrovascular disease.

### Prevalence of NAFLD and suspected advanced fibrosis at FU (Fig. 1)

Among the 1380 PLWH included in the FU analyses, NFS was provided in 1283 (93.0%). In 1039/1283 (81.0%) subjects advanced fibrosis was ruled out according to NFS < −1.455. 21 (1.6%) individuals showed NFS scores ≥ 0.676, indicating advanced fibrosis, while intermediate NFS scores between ≥ −1.455 and < 0.676 resulted in 223 (17.4%) cases.

Metabolic comorbidities were documented in 20 (95.2%) of the 21 PLWH with suspected advanced fibrosis according to NFS at FU (BMI ≥ 25.0 kg/m^2^: *n* = 15 (71.4%), dyslipidemia: *n* = 10 (47.6%), DM: *n* = 10 (47.6%), hyperuricemia: *n* = 7 (33.3%), arterial hypertension: *n* = 11 (52.4%), cardiovascular disease: *n* = 2 (9.5%)). Only 1/21 PLWH with suspected advanced fibrosis showed no signs of metabolic dysfunction at BL or FU and was never treated with PI-containing ART regimens. However, while dyslipidemia had not been diagnosed in this individual, specific lipid profile data (serum HDL, LDL, triglycerides) were not available for BL evaluation. None of the PLWH with suspected advanced fibrosis had cerebrovascular disease at FU and 9/21 (42.9%) subjects were treated with PI-containing ART regimens at FU.

Among the 21 PLWH with advanced fibrosis at FU, 5 (23.8%) showed AST, ALT and GGT levels within the normal range; one of them was the patient who showed an unremarkable metabolic profile at FU.

### Mortality among the study population

During a median follow-up of 11.7 (IQR 11.7) years, 54 (3.7%) of the 1458 PLWH included in the study population died: 18 (33.3%) died due to HIV-related reasons including HIV-associated malignancies, 7 (13.0%) due to cardiac, cerebrovascular, and metabolic complications, 6 (11.1%) due to substance-related adverse events and intoxication, 5 (9.3%) due to hemato-oncological reasons, 2 (3.7%) due to liver-related events including primary liver cancer, 1 (1.9%) due to suicide, and 15 (27.8%) due to other reasons. Of the 54 PLWH who died during follow-up 7 (13.0%) had suspected advanced fibrosis according to NFS ≥ 0.676 at last FU; in 1 (14.3%) of the 7 subjects with suspected advanced fibrosis the reported cause of death was liver-related (hepatocellular carcinoma).

## Discussion and conclusion

Due to the availability of modern ART regimens, the AIDS-related mortality has declined significantly, which in turn unmasks other health challenges PLWH are facing [13, 20]. The aim of this study was to describe the epidemiology of NAFLD-associated liver disease and metabolic risk factors in our Viennese PLWH cohort.

Metabolic comorbidities were present in 53.8% of PLWH at BL and this already high rate increased to 68.1% at FU. Of note, 34.7% of the patients had dyslipidemia at BL, 11.7% of which were on lipid-lowering therapy, and after a follow-up of 6.3 years, the rate of dyslipidemia increased to 42.8% and the uptake of lipid-lowering treatment also increased to 43.7%. Due to the timing of the retrospective data collection finished in June 2017, the respective ESC definition of dyslipidemia was used by then. However, current guidelines set considerably lower LDL cut-offs for the definition of hyperlipidemia and, hence, for the indication of lipid-lowering drugs [30]. When applying the definitions according to the 2019 ESC/EAS guidelines for the management of dyslipidemias, the percentage of PLWH with dyslipidemia among our study population would be as high as 43.6% at BL and 50.8% at FU [30]. Importantly, the percentage of PLWH with dyslipidemia among those with suspected advanced fibrosis would increase to 56.3% at BL and 57.1% at FU, according to the 2019 ESC LDL cut-offs. Due to the retrospective character of the study, it cannot be warranted that all blood samples derived from fasting individuals; therefore, triglyceride levels were only displayed in the patients’ characteristics table but were not used for the retrospective diagnosis of dyslipidemia [29]. Similarly, fasting glucose levels were not taken into consideration, even though they are among the criteria suggestive of NAFLD according to the 2016 EASL-EASD-EASO clinical practice guidelines for the management of non-alcoholic fatty liver disease [29]. However, previously diagnosed diabetes and elevated HbA1c were evaluated to assess the prevalence and incidence of DM among our study population.

The high rate of dyslipidemia in our study population is especially relevant as PLWH are known to have an increased risk for the development of cardiovascular events as compared to HIV-negative subjects, which is further increased by the initiation of ART [31]. While the overall benefits of ART certainly outweigh the potential increase in cardiovascular risk in PLWH, monitoring and optimization of metabolic and cardiovascular risk factors are imperative in this special population. Importantly, PLWH are considered patients at high risk for cardiovascular disease according to the ESC, therefore the corresponding LDL target values should be aimed for when treating PLWH for dyslipidemia [31]. While positive effects of statins on liver disease have been demonstrated previously, drug-drug interactions with ART need to be considered carefully in PLWH [32]. Among our study population, only 39.0% and 39.9% of the patients showed LDL levels within the recommended range for PLWH at BL and FU, respectively, while 95.1% and 83.2% patients remained without any lipid-lowering medication despite their LDL levels exceeding the recommended cut-off of 100 mg/dL [31].

While increased waist circumference is considered another risk factor suggestive of NAFLD [29], it could not be directly assessed in this retrospective analysis. However, BMI was used as a surrogate parameter in our study population. At BL, the majority of PLWH included in our study were of normal weight or overweight (87.1%) and only 7.1% were considered obese according to BMI. Interestingly, 5.9% PLWH showed underweight at BL, which may be attributable to the high frequency of AIDS stage HIV infection among our study population. During the observation period, BMI increased substantially with only 56.2% of PLWH being underweight or normal weight at FU while 13.6% were obese. The percentage of hyperuricemia (10.8% vs. 11.2%) and cerebrovascular disease (0.5% vs. 0.7%) remained relatively stable between BL and FU, yet there was a notable increase in arterial hypertension (6.6% vs. 26.8%), DM (1.4% vs. 4.7%) and cardiovascular disease (1.4% vs. 5.0%). This goes in line with the known increased cardiovascular risk of PLWH [31] and may additionally be fuelled by the ageing process during FU.

Despite the high rate of metabolic comorbidities among our study population, only 1.3% and 1.6% showed signs of advanced fibrosis according to NFS at BL and at FU, respectively [19, 25, 26]. However, we found a high percentage of PLWH with intermediate NFS, who by definition of the score, would need further assessment via imaging, elastography and/or liver biopsy [26]. Importantly, 72.3% (BL) and 89.2% (FU) PLWH with intermediate NFS had metabolic comorbidities. Hence, we believe that there may be a relevant number of NAFLD cases among our study population that remained undetected by NFS.

Overall, our study population consisted primarily of subjects who acquired HIV infection through sexual practices among MSM (778/1458, 53.4%). This is interesting as HIV remains common in PWID; however, a relevant number of PWID living with HIV had previously been excluded from the study due to viral hepatitis coinfection [33, 34]. Of note, there were no PWID among those with suspected advanced fibrosis according to NFS at BL, which may be attributable to the lower prevalence of metabolic comorbidities (45.8% vs. 54.3% among non-PWID) and the slightly lower BMI (median 22.6 kg/m^2^ among PWID vs. 23.3 kg/m^2^ among non-PWID). Furthermore, only 59.0% of the PWID included received ART at BL, among which 59.2% were on PI-containing regimens vs. 35.1% of the 67.7% who were on ART received a PI among the non-PWID population. On the other hand, the fact that there were also no patients with NFS ≥ 0.676 among those with suppressed or missing HIV-RNA at BL may be associated with the prosteatotic effect of HIV-induced immune activation [20]. Overall, the small number of cases (BL 16/1272, 1.3%) with suspected advanced fibrosis among our cohort does not allow for solid conclusions. However, the low rate of suspected advanced fibrosis along with the availability of specialized HIV care in the setting of a large tertiary care hospital may support the low rate (0.1%) of liver-related deaths among our study population.

While we consider the large sample size of 1458 PLWH in whom viral hepatitis coinfection was ruled out as a major strength of this study, it also has several limitations. The most relevant limitation is the lack of invasive or imaging proof of steatosis or steatohepatitis. While liver biopsy remains the gold standard for the diagnosis of NAFLD and represents the only tool for differentiation between steatosis and steatohepatitis, imaging techniques and transient elastography are widely available noninvasive alternatives for the diagnosis of NAFLD and ultrasound is an easy to use tool for primary evaluation of steatosis [29, 35]. However, none of these parameters, including transient elastography and alternative noninvasive tests to evaluate fibrosis, like the Fibrosis-4 Index, are comprehensively available for our study population, therefore NAFLD fibrosis was solely evaluated by NFS [19, 26, 35]. This is mainly attributable to the fact that the patients included in this study were primarily managed at the HIV clinic of our institution, where liver stiffness measurements are not routinely performed. Nevertheless, a total of 28/1380 PLWH without pre-existing or incident viral hepatitis coinfection who were included in the analyses of our study underwent transient elastography (FibroScan; Echosens, Paris, France) at BL and/or at FU and showed a significant correlation between liver stiffness measurement by transient elastography and NFS (R = 0.525, *p* = 0.004), which was also confirmed in an independent group of 92 patients with biopsy-proven NAFLD and without HIV infection (R = 0.495, *p* < 0.001) (Supplementary figures 1–2). While these results suggest a strong correlation of NFS with liver stiffness in our cohort of PLWH, the true rate of NAFLD/NASH fibrosis remains to be established, especially in patients with intermediate NFS in whom advanced fibrosis cannot be ruled out with sufficient sensitivity. The retrospective design of the study represents another limitation and explains that several important laboratory parameters, such as fasting blood glucose and triglyceride levels as well as clinical parameters like waist circumference cannot be provided [10, 29].

However, we still believe that this study shows important epidemiological data on NAFLD-associated liver fibrosis in PLWH. Interestingly, the longitudinal analyses of the metabolic profiles in the included PLWH indicate dynamics of dyslipidemia and diabetes over time, and suggest that exposure to PI-containing ART promotes advanced NAFLD fibrosis.

## Supplementary Information

Supplementary figure 1

Supplementary figure 2

Supplementary figure legends
