# Supplementary figures and images for: Prevalence and dynamics of NAFLD-associated fibrosis in people living with HIV in Vienna from first presentation to last follow-up
Source: Wien Klin Wochenschr. 2022 Dec 28;135(15-16):420–8. doi: 10.1007/s00508-022-02133-9 (PMC10444631; doi:10.1007/s00508-022-02133-9)

Supplementary figure 1

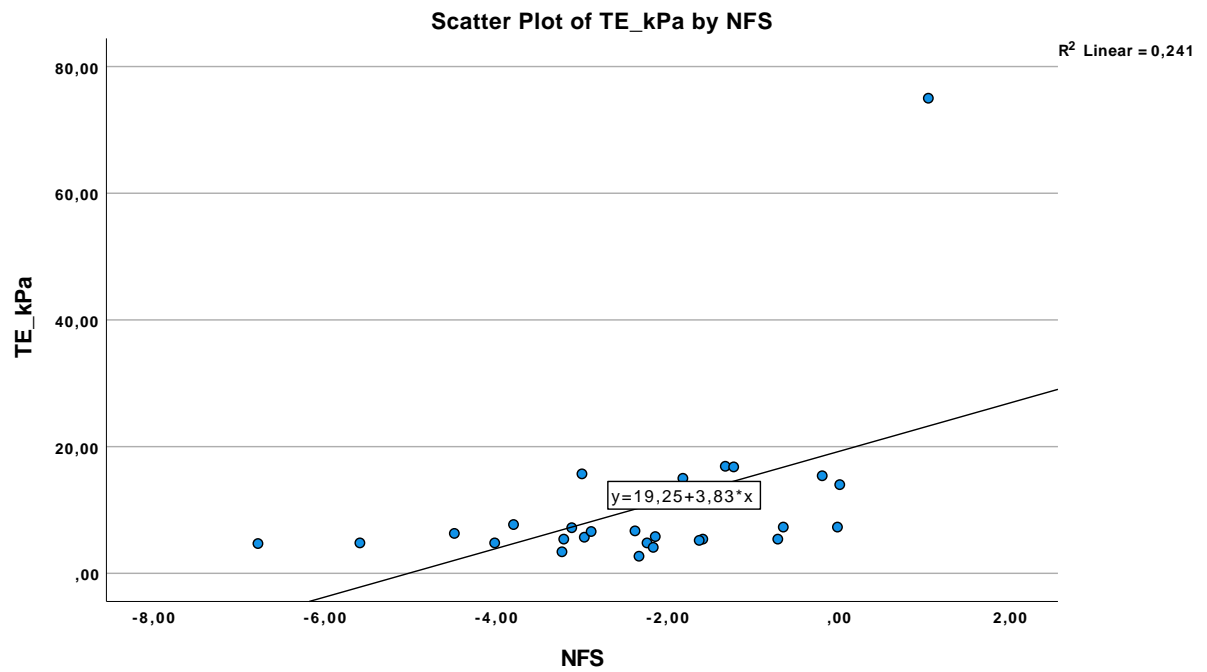

Supplement: Supplementary file 1 — Supplementary figure 1 [file 508_2022_2133_MOESM1_ESM.pdf]

Supplementary figure 2

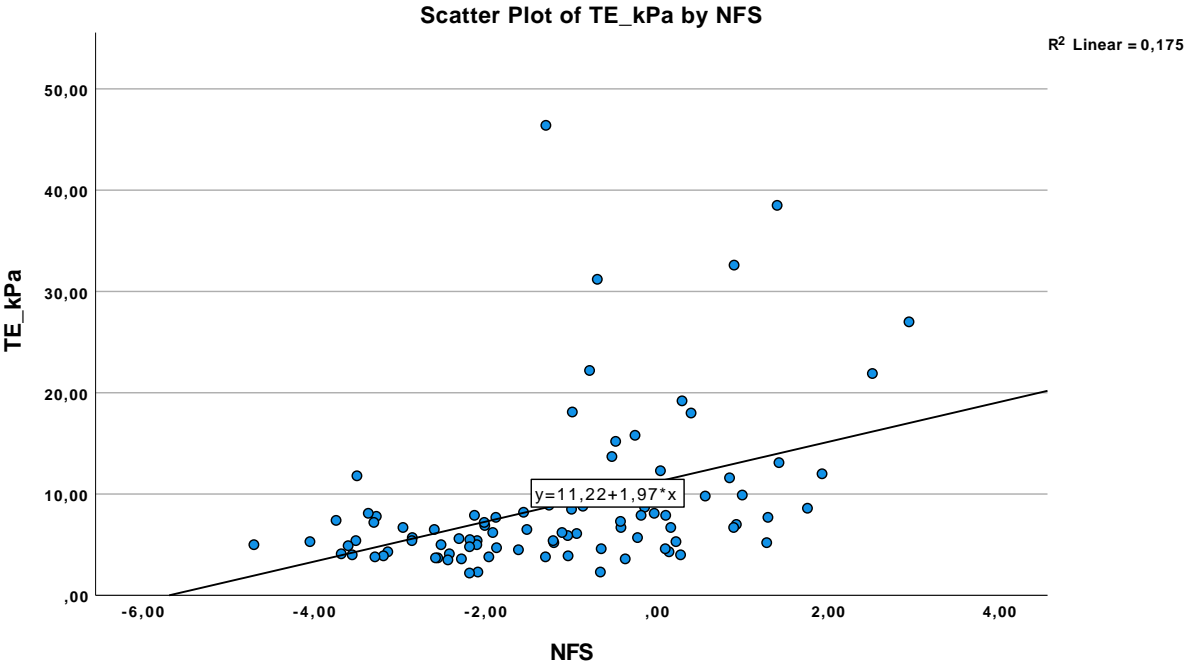

Supplement: Supplementary file 2 — Supplementary figure 2 [file 508_2022_2133_MOESM2_ESM.pdf]
