# Supplementary material for: Prevalence and dynamics of NAFLD-associated fibrosis in people living with HIV in Vienna from first presentation to last follow-up
Source: Wien Klin Wochenschr. 2022 Dec 28;135(15-16):420–8. doi: 10.1007/s00508-022-02133-9 (PMC10444631; doi:10.1007/s00508-022-02133-9)
Supplement: Supplementary file 3 — Supplementary figure legends [file 508_2022_2133_MOESM3_ESM.docx]

**SUPPLEMENTARY MATERIAL**

**Supplementary figure 1: Correlation between NAFLD-fibrosis score and liver stiffness according to transient elastography in PLWH.**

28 PLWH included in the analyses of this study had TE results available at BL and/or at FU. Among this sample, Spearman's rank correlation analysis revealed a significant correlation between NFS and liver stiffness according to TE (R = 0.525, p = 0.004).

Abbreviations: TE, transient elastography; TE_kPa, transient elastography measured in kPa; NFS, NAFLD-fibrosis score; PLWH, people living with HIV; BL, baseline; FU, follow-up.

**Supplementary figure 2: Correlation between NAFLD-fibrosis score and liver stiffness according to transient elastography in individuals with biopsy-proven NAFLD.**

Among an independent sample of 92 subjects with biopsy-proven NAFLD and without HIV-infection, Spearman's rank correlation analysis confirmed a significant correlation between NFS and liver stiffness according to TE (R = 0.495, p < 0.001).

Abbreviations: TE, transient elastography; TE_kPa, transient elastography measured in kPa; NFS, NAFLD-fibrosis score.
